# The Role of Physical Education in Preventing Unhealthy Lifestyles in Immigrant Adolescents

**DOI:** 10.3390/ijerph19116889

**Published:** 2022-06-04

**Authors:** Juan-Antonio Mondéjar-Jiménez, Guillermo Ceballos-Santamaría, Andrés Valencia-García, Francisco Sánchez-Cubo

**Affiliations:** Faculty of Social Sciences of Cuenca, University of Castilla-La Mancha, 16071 Cuenca, Spain; juanantonio.mondejar@uclm.es (J.-A.M.-J.); guillermo.ceballos@uclm.es (G.C.-S.); andresvagar@hotmail.com (A.V.-G.)

**Keywords:** education, health, healthy lifestyles, immigrant teenagers, physical activity

## Abstract

In recent years, migratory phenomena have changed the composition of Spanish society. There are many studies that explore the healthy habits of young adolescents, but few focus on young immigrants. The purpose of this study is to examine the causal relationships between certain factors that influence the health of immigrant youth and sports. The sample consisted of 173 students enrolled in secondary education in the city of Cuenca. The structural model confirms the relationship between the latent variables and sports. Specifically, we obtained a positive influence of an active lifestyle (regular physical activity and exercise) and a negative influence for the remaining variables (alcohol consumption, unhealthy foods, self-medication and tobacco consumption in the family). Physical education should promote healthy lifestyles; greater coordination between physical education and other subjects involved in education and the promotion of health are necessary because we consider that this task is not exclusive to physical education.

## 1. Introduction

During recent years, Europe has been experiencing a steady increase in immigrant flows from inside and outside EU countries, but despite their importance to the society, their migration flows remain an understudied phenomenon when it comes to immigration’s interrelations with other issues and its implications. These flows entail noticeable consequences that should be properly addressed to favour the integration of immigrants and in turn enrich the host societies. That is also the case for education, especially at earlier education levels in which addressing multiculturalism—now overcome by the idea of interculturality—is key to allowing young immigrants to fully develop themselves. According to Banks [1], interculturality tries to preserve and extend cultural diversity, attempting to promote the equality of economic, social and political possibilities by encouraging the acquisition of cultural competencies, which allows each person to use them in different cultural contexts. An intercultural school is a prior and necessary step for an intercultural society.

In this context, the cohabitation established between immigrant and native populations has been widely studied, thereby providing explicative models of the reality rather than ideal models of coexistence. Otherwise, the sociological studies taken during the 21st century in America that responded to the possible ways of adding minority groups to a previously settled majority society have aimed to explain the relationships between immigrants and the native populations [2].

All these and many more studies have widely contributed to the scientific literature, providing very interesting approaches and dimensions to study. Considering that fact, this work aims to address a particular gap which remains slightly understudied in the Spanish case: young immigrants and their relationship to physical activity within the Spanish educational system.

Moreover, this topic is of great interest given the increases in immigrant flows that have occurred during the last years and the ones that are expected in the following decades. Regarding the extant data, the national figures show a steady decrease of the Spanish population while there is a noticeable increase in the number of immigrants, which helps sustain the population levels of the country. Specifically, in July 2021, there were 47,326,687 people living in Spain, of whom 7,219,701 were foreigners. In other more illustrative words, since January 2016 to July 2021, the Spanish population grew 1.91% but in the same period, the Spanish-born citizenry decreased by 1.02% while the immigrant population rose by 21.99% [3]. This is also applicable to the city of Cuenca, where this study takes place. The latest available data at the municipal level are from 2008, provided by the Social Intervention Area of the City Council of Cuenca through the Intercultural Care and Mediation Service [4] and not publicly available. These data showed that 10.1% of the population of Cuenca is immigrant, of whom 9.7% are non-EU citizens or recently included, and 0.4% are from EU and developed countries. The most updated data are at the province and city levels, but these are provisional data that do not yet differentiate between Spanish and immigrants [5]. However, the data do illustrate the problem of depopulation: from 2016 to 2021, the province has lost 2.76% of its population and the city of Cuenca, 2.02%. Therefore, this case study is especially interesting because it is of an inland city which suffers from depopulation. An increase in the number of immigrants is of high interest, even more, young immigrants since they may contribute to slowing down depopulation among other benefits. In addition, this study is very representative and provides insightful data and results since the sample is almost a census sample (173 answers obtained out of 266 immigrant students in the city). Therefore, the results should be solid for enhancing informed decision-making and public policies.

Caring about young immigrants’ integration, acculturation, health and well-being should be on public agents’ agendas. These topics have been addressed in the literature for adults [6,7,8], children [9,10,11], adolescents [12,13] and households [14]. However, this work focuses only on studies on young immigrants, analysing the underlying dimensions of a healthy lifestyle—exercise, physical activity and nutrition, among others—and their effects on practising sports. This differentiation with respect to local students is made because the literature has already found that immigrants adopt unhealthier habits when they move to the destination country [15] and that these effects also impact their children, who end up having worse health and habits than their local counterparts [11,12,13,14,16,17,18]. In this sense, it is important to mention that although the majority of studies found differences between locals and immigrants at different stages, some have found few or no differences in physical [11], physiological or behavioural [19,20] activity.

In short, healthy lifestyles have been studied from multiple approaches: from explicative models of the socialisation process to expectancy-value theories; from theories on the self-regulation of behaviour to models based on the balance theory of decision-making. In addition, the negative effects of physical inactivity and the health benefits provided by systematised physical activity have been broadly documented. For this reason, the programmes of health promotion worldwide have usually been based on the integration of physical activity in daily life by encouraging all social groups through intervention strategies [21]. The positive relationship between physical activity and health is widely known [22,23,24,25], but the theoretical models or paradigms which study these relationships are continuously revised and transformed. There partially lies much of the importance of this work.

Furthermore, many research projects attempt to explain the main motivations for young people to practise sport. Some of them state that one of the main reasons for practising sports is the enjoyment of physical activity, and others include the pursuit of good physical condition [26] and exercise. Therefore, we consider physical activity a factor that positively influences sports practice, and so a positive relationship between practising physical exercise and sport is expected.

Negative effects on the likelihood of practising sports are analysed as well. Some studies suggest a negative influence of teenage alcohol [27,28] and tobacco [29] consumption on sports practice. We also expect to find a negative influence of the consumption of unhealthy foods on sports practice, following numerous studies that point to a direct relationship between physical exercise and balanced nutrition and how it is possible to influence other health variables such as nutrition acting on physical practice [14,30]. Finally, a few studies address the relationships between self-medicated drugs and physical activity. Their results generally show a negative influence [31], so we expect to find in our study the same relationship.

In essence, this work is included in the line of research on integrative models; that is to say, it analyses the variables which influence the healthy lifestyles of a sample of young immigrants for guiding future works on the adaptation of methods, programmes, workshops, seminars and/or activities aimed at influencing even healthier lifestyles. These activities should be aimed at adolescents but also at their parents since their environments may also affect these dimensions [7,11,32,33]. Therefore, the objective of this work is to establish causal relationships between sports practice and healthy lifestyles in adolescent immigrants. The hypotheses are as follows:

**Hypothesis** **1** **(H1).** *There is a relationship between sports practice and a healthy lifestyle in the assessed population.*

**Hypothesis** **2** **(H2).** *The practice of physical activity positively influences sports practice.*

**Hypothesis** **3** **(H3).** *The consumption of alcohol, tobacco, unhealthy foods and self-medicated drugs has a negative impact on sports practice.*

## 2. Materials and Methods

The data collection tool used was an adaptation of the Inventory of Health Behaviours in School-Age Children [34], designed for assessing different variables of a healthy lifestyle. The European Regional Bureau of the World Health Organization adopted it within the framework of an international investigation of the health habits of young people [35], which has made it the most commonly used tool for this purpose in Europe but also worldwide [36]. Table 1 shows the indicators analysed and their explanations.

In Spain, the inventory has been adapted and used in multiple studies [37]. It stands out that the tool is consistent with the cultural differences of immigrants. That is because of the design of the survey itself—intended for transcultural studies—and because respondents complete the survey in small, supervised groups, which allow a person in charge to solve doubts.

The original purpose of this study was census-based data collection. Thus, surveys were sent to the 266 immigrant students of Cuenca enrolled in the secondary schools. Since one of the secondary schools did not deliver the surveys, the final sample was 173, which is the total number of immigrant students who attend classes in the rest of the secondary schools. Consequently, the sample is a convenience sample. Considering the total parameters, we obtain error rates lower than 5% (4.4%) for a confidence level of 95%. The census-based nature of this study is the main reason for these low error rates, so we consider that the study sample is representative.

The methodology used was the structural equation model (SEM), which is a multivariate statistical analysis method applied for contrast models that propose causal relationships between variables [38,39,40]. It is widely used in multiple works which have established explicative models of the influences of certain variables on health in adolescents: in terms of physical activity [29,41,42]; alcohol consumption [43,44,45]; tobacco consumption [46]; nutrition [42] and education [47,48] as well as medical use.

A similar situation occurs, from many diverse perspectives, in immigration. Within this field, we can find, among others, studies that refer to intercultural relations between immigrants and natives and gender differences between immigrants [49].

The model we propose establishes causal relationships between some of the variables which determine the healthy behaviour of adolescents. It considers seven adolescents’ health conditioning factors as latent variables, and the survey answers are the indicators. The estimations were performed using SmartPLS 2.0.M3 software [50].

## 3. Results

The results obtained for the model represent a measure of the validity of the survey used for capturing the seven latent dimensions through indicators which were verified. The usual measure of goodness of fit, proposed by Tenenhaus et al. [51], is the geometric mean of the average communality (external model) and the average R^2^ (internal model), with a value of 0.512. Concerning the reliability of the measuring tool (Table 2), the value of Cronbach’s alpha for all the latent variables is higher than or very close to 0.7 [52]. The composite reliability scores are also higher than 0.8 in all cases. Concerning the convergent validity (AVE), the values of the seven dimensions are higher than or very close to 0.5, as recommended by Fornell and Larcker [53]. Likewise, cross-loadings are always higher for the latent variables in which the respective elements are loaded (Table 3).

**Figure 1:**
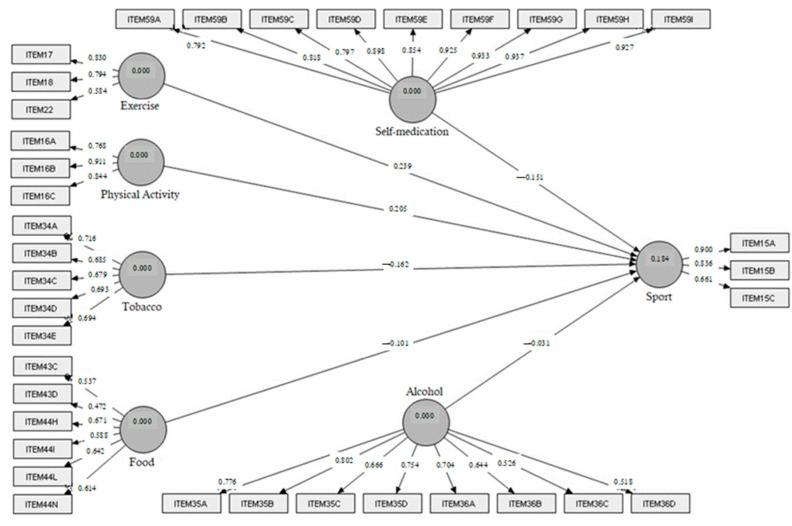
The influence model of healthy habits and sports practice.

With the data obtained, we propose the first model (Figure 1), which refers to the causal relationships of six variables that influence the sports practice of immigrant adolescents.

The discriminant validity criterion [53] is also applied for the seven latent variables. The respective AVE is higher than the square of the estimated correlation between them (Table 2).

AVE_i_ > p^2^_ij_

Regarding the structural model, as shown in Figure 1 and Table 4, the R^2^ coefficients associated with the regressions of the latent variables are statistically significant, with values higher than 0.1 obtained in all cases [54]. An analysis of the direct effects represented in Table 3 highlights the dependency between the latent variables and confirms the initial assumptions of the model. As all values are different from 0, the relationship of dependence between sports practice and the rest of the latent variables is demonstrated. Further analyses will be made in the discussion section.

Summing up all the above, the results revolve around the proposal, assessment and validation of a PLS-SEM model. The model (Figure 1) has six constructs which represent the dimensions that are likely to affect sports. These constructs are made up of specific indicators (Table 1), all of them representative of each latent variable since their AVE, composite reliability and Cronbach’s alpha values (Table 2) are above the required thresholds in almost all cases. Once the constructs are assessed, their paths to the dependent construct (sports) are analysed in Table 4. Since these are all different from 0, the relationships can be established. In this sense, the signs and sizes of these effects are of high relevance. Only physical activity (0.2054) and exercise (0.2386) have positive impacts on sports, while the rest negatively affect it. All these constructs and their relationships help in explaining sports through R^2^, which is above the threshold (0.184). Lastly, Table 3 deepens the correlations between the constructs, showing their types and sizes. The interpretation of this matrix is suggested to be performed following Cohen’s [55] criterion, which states the thresholds as small, <0.3; medium, <0.5; and large, >0.5. Considering this criterion, the correlation matrix contains 18 small, 2 medium and one large correlation. The practical consequences are discussed in the following section.

## 4. Discussion 

It seems crucial to understand students to plan pedagogic programs suitable for their needs from any perspective. In this context, physical education courses should promote healthy lifestyles, not only focused on the body but on other variables of lifestyle which influence sports practice. The law that establishes the secondary education curriculum in Castilla-La Mancha includes aspects addressing health and physical condition, such as the importance of breakfast and a balanced diet, the negative effects of certain foods and substances and the positive effects of physical activity on the human body. In practice, however, these points have a low teaching load compared with other practical aspects like sports.

In the results section, the positive influence of some variables in sports practice is shown. The most notorious are those more related to the dependent variable construct because of their conceptual proximity to both variables. Thus, the direct relationships between physical exercise (0.239) [11,12,13,14,16] and physical activity (0.205) [22,23,24,25] with sports stand out, the former being the most relevant of all the variables. This can be explained by the relationship between both manifestations of human motor activity, which results in a positive tendency among those who practise any ordered and systematised physical activity, entailing a step forward in the regulation and institutionalisation of human motor activity. Additionally, the influence of physical activity on sports practice (0.205) is noticeable, which means that active habits in daily life are extended in the systematised, regulated and institutionalised practice that characterises sports. Considering some descriptive figures from the questionnaire, Spain stands out as a sedentary country. Only 36.3% of the immigrant population analysed perform physical activity twice or three times a week with moderate activity (70%) for more than 45 min (41.8%). Sports experiences a slight increase to 38.6%, but 30.7% never practise sports or do not regularly. Consequently, we predict that if physical education classes encourage an active daily life that may favour adopting the practice of physical exercise or participation in physical activities, teachers would positively influence students to join sports activities.

Conversely, the inverse relationships between the consumption of certain substances such as alcohol (−0.0301) [27,28], unhealthy food (−0.101) [14,30] and self-medicated drugs (−0.151) [31] and familial tobacco consumption (−0.162) [29] and sports lead us to consider the need for greater coordination between physical education and other subjects involved in the promotion of health because we consider that this task is not exclusive to physical education. In this sense, tutorial action in education centres is needed, and it is formalised through the tutorial action plan, understood as a framework paper that gathers the organisation and functioning of the conducted mentoring. In the case of secondary education, it is prepared by the guidance department. It would be of interest to use the obtained results for programming workshops about acquiring good nutritional habits and avoiding alcohol and tobacco consumption, not only because of their negative impacts on sports practice but also their negative impacts on health.

Among these, the higher intensity found in tobacco consumption stands out. Although it cannot be confirmed with certainty, due to the effects that tobacco produces on passive smokers, remarkably, it is the only drug that acts not only on the consumer but also on others. This situation motivates promoting sports practice through intervention in the family environment and not only with a given person. The effects of these four negative constructs go as follows.

First, familial tobacco consumption has a negative effect on sports practice (−0.162) [29], which emphasises the effect on the individual’s own behaviour, in this case the sports practice. However, it is also striking that 7.6% of the students smoke daily and 5.3% weekly, although not all days. Moreover, the preferred substance for smoking is tobacco, but others such as hashish and joints are usually smoked by 7.6% of the students. Second, the relationship between self-medication and sports activities is reverse (−0.151) [31]. The unwise variable self-medication, negative itself, also acts as a negative influence on acquiring healthy behaviours such as sports practice. In this sense, it is worrying that between 27.4% and 37.6% self-medicate against colds, headaches or coughs. Despite representing a low percentage, some adolescents need tranquillisers (8.3%) or sleeping pills (4.8%). Then, the consumption of unhealthy foods has a negative impact on sports practice (−0.101) [14,30]. That could be interpreted in two ways because those who practise sports also have a healthier diet; hence health effects grow. Lastly, alcohol consumption has a negative effect on sports practice (−0.031) [27,28]. The effects of alcohol, and also probably the contexts in which young people drink alcohol (with friends, in their free time), have a negative influence on sports practice. Indeed, 38.6% claim to have been drunk at least once, and 5.6% more than ten times. The favourite type of alcohol for young people is beer, followed by cocktails, wine and cava.

## 5. Conclusions

Considering all the above, this study showed that some lifestyle habits related to the culture such as diet and alcohol, tobacco and drug consumption create an intercultural relationship context that has already been proven to affect immigrant individuals [8,11,32]. Furthermore, as we noticed in earlier studies, there are no significant differences, so it is possible to use this field for promoting equality through education. Regarding the nutrition issue, low energy consumption combined with inadequate nutrition potentially results in young overweight and obese populations. When analysing nutrition, we obtained some interesting and contradictory data: 44.7% of the respondents believe they have a healthy diet, while 25% admit to drinking carbonated beverages daily, and 17.8% eat sweets daily. These figures may seem low but are combined with the low consumption of vegetables (25.3%) and fruits (55.1%), an excess of dairy products (57.4%) to the detriment of other sources of protein such as fish or meat, and the abuse of carbohydrates (68.9%). Thus, nutritional deficiencies in young people arise, and performing awareness and prevention campaigns seem necessary.

Therefore, physical education teachers—but also educational institutions with public administration’s support—should raise awareness of the importance of a healthy diet, with lower consumption of saturated fats, hamburgers, cold meats, chips, et cetera. We should also continue with the campaigns carried out in the base education on healthy breakfasts (only 46.9% of girls have breakfast) or the importance of having five meals a day (only 30% of boys and 5.9% of girls have them).

All in all, this paper provides further data and analyses in the field of health habits and sports in young immigrants. Many relationships are shown, and suggestions are proposed along with them. However, this study presents limitations as it is focused on an inland Spanish medium-sized city. Nevertheless, it contains useful results to be applied by teachers, schools and institutions to favour a better integration of young immigrants, providing them with the resources to tackle unhealthy habits in this minority group. In this sense, the future lines of research revolve around the actualisation of the information contained in this work, considering new contextual dimensions such as nationality, size of the city and changes in legislation. Changes in immigration flows might also affect young people’s lifestyle habits, so the authors should check whether changes occur in the following years.

## Figures and Tables

**Table 1:** The constructs and indicators used in this study.

Construct	Item	Measure (Likert Scale)	Description
Sports	15A	1 to 6	Frequency
	15B	1 to 4	Intensity
	15C	1 to 6	Duration
Physical Activity	16A	1 to 6	Frequency
	16B	1 to 4	Intensity
	16C	1 to 6	Duration
Exercise	17	1 to 7	Weekly frequency in days
	18	1 to 6	Weekly frequency in hours
	22	1 to 3	Exercise alone
Tobacco in families	34A	1 to 4	Intensity of consumption (father)
	34B	1 to 4	Intensity of consumption (mother)
	34C	1 to 4	Intensity of consumption (elder brother)
	34D	1 to 4	Intensity of consumption (elder sister)
	34E	1 to 4	Intensity of consumption (best friend)
Alcohol	35A	1 to 2	Drinks beer (1 yes, 2 no)
	35B	1 to 2	Drinks wine (1 yes, 2 no)
	35C	1 to 2	Drinks cocktail or liqueur (1 yes, 2 no)
	35D	1 to 2	Drinks cava (1 yes, 2 no)
	36A	1 to 5	Intensity of beer consumption
	36B	1 to 5	Intensity of wine consumption
	36C	1 to 5	Intensity of cocktail or liqueur consumption
	36D	1 to 5	Intensity of cava consumption
Food	44C	1 to 4	Frequency of drinking sodas
	44D	1 to 4	Frequency of eating sweets
	44H	1 to 4	Frequency of eating chips
	44I	1 to 4	Frequency of eating burgers or sausages
	44L	1 to 4	Frequency of eating cold cuts
	44N	1 to 4	Frequency of eating butter
Self-medication	59A	1 to 3	Frequency of self-medication for cough
	59B	1 to 3	Frequency of self-medication for cold
	59C	1 to 3	Frequency of self-medication for headache
	59D	1 to 3	Frequency of self-medication for stomachache
	59E	1 to 3	Frequency of self-medication for difficulty sleeping
	59F	1 to 3	Frequency of self-medication for nervousness
	59G	1 to 3	Frequency of self-medication for tiredness
	59H	1 to 3	Frequency of self-medication for constipation (laxatives)
	59I	1 to 3	Frequency of self-medication for lose weight

Source: Authors.

**Table 2:** The reliability of the measuring tool.

	AVE	Composite Reliability	R Square	Cronbach’s Alpha	Communality	Redundancy
PA (Physical Activity)	0.7106	0.88		0.7936	0.7106	
Alcohol	0.4643	0.8715		0.8614	0.4643	
Food	0.34	0.753		0.6218	0.34	
Self-medication	0.7902	0.9712		0.9664	0.7902	
Sports	0.6492	0.8453	0.1836	0.7743	0.6492	0.0374
Exercise	0.5328	0.1553		−0.7869	0.5328	
Tobacco in families	0.4811	0.8225		0.7324	0.4811	

Source: Authors.

**Table 3:** The correlation matrix between the latent variables.

	PA (Physical Activity)	Alcohol	Food	Self-Medication	Sport	Exercise	Tobacco in Families
PA (Physical Activity)	1.0000						
Alcohol	−0.0076	1.0000					
Food	0.1115	0.0958	1.0000				
Self-medication	0.0788	−0.0177	0.0604	1.0000			
Sports	0.2133	−0.1159	−0.1375	−0.1538	1.0000		
Exercise	0.2112	−0.0704	−0.0781	−0.0806	0.3054	1.0000	
Tobacco in families	0.1214	0.3664	0.1795	−0.036	−0.1626	−0.0075	1.0000

Source: Authors.

**Table 4:** The effects of the latent variables on sports.

	Sport
PA (Physical Activity)	0.2054
Alcohol	−0.0314
Food	−0.1006
Self-medication	−0.151
Sports	
Exercise	0.2386
Tobacco in families	−0.1616

Source: Authors.

## Data Availability

The data are not publicly available due to the nature of the study.
